# Activated Schwann cells and increased inflammatory cytokines IL‐1β, IL‐6, and TNF‐α in patients' sural nerve are lack of tight relationship with specific sensory disturbances in Parkinson's disease

**DOI:** 10.1111/cns.13282

**Published:** 2019-12-11

**Authors:** Hui Zhang, Jing Wu, Fei‐Fei Shen, Yong‐Sheng Yuan, Xiao Li, Pan Ji, Lin Zhu, Li Sun, Jian Ding, Qi Niu, Ke‐Zhong Zhang

**Affiliations:** ^1^ Department of Neurology The First Affiliated Hospital of Nanjing Medical University Nanjing China; ^2^ Department of Pathology The First Affiliated Hospital of Nanjing Medical University Nanjing China; ^3^ Department of Neurology The First People's Hospital of Changzhou Changzhou China

**Keywords:** neuroinflammation, Parkinson's disease, peripheral nerve, schwann cells, sensory disturbance

## Abstract

**Aims:**

Neuroinflammation is one of the most important processes in the pathogenesis of Parkinson's disease (PD). Sensory disturbances are common in patients with PD, but the underlying pathophysiological mechanisms remain unknown. This study aimed to characterize the activation of Schwann cells (SCs) and the increase of expression of inflammatory cytokines IL‐1β, IL‐6, and TNF‐α in the sural nerve of PD, and further explore whether peripheral nerve inflammation is the cause of PD sensory disturbances.

**Methods:**

A total of 14 patients with PD (including 5 with sensory disturbances and 9 without sensory disturbances) and 6 controls were included. The excitation and conduction function of sural nerve was detected by sural nerve electrophysiological examination. With sural nerve biopsy samples, ultrastructural changes of sural nerve were observed by electron microscopy; Schwann cell biomarker glial fibrillary acid protein (GFAP) and inflammatory cytokines including interleukin‐1beta (IL‐1β), interleukin 6 (IL‐6), and tumor necrosis factor‐alpha (TNF‐α) were detected by immunohistochemistry, and the outcome of immunostaining slice was semiquantitatively counted; double immunofluorescence was used to identify the locus immunoreactive for inflammatory cytokines.

**Results:**

Compared with healthy controls, nerve conduction velocity (NCV) slowed down and sensory nerve action potential (SNAP) amplitude decreased in PD patients, accompanied by axonal degeneration and demyelinating lesions, and expression of GFAP and inflammatory cytokines was increased. Inflammatory cytokines were significantly colocalized with GFAP and slightly colocalized with NF. These indicators did not differ significantly between PD patients with and without sensory disturbances.

**Conclusion:**

Our study results suggest that peripheral sensory nerve injury exists in PD patients, accompanied by Schwann cell activation and inflammation, thus demonstrate peripheral nerve inflammation participates in the pathophysiological process of PD but it is not necessarily related to the patient's sensory disturbance.

## 1 INTRODUCTION

It is estimated that 30%‐85% of patients with PD suffered from sensory disturbances such as pain, numbness and paresthesia.^1^, ^2^ It has been pointed out that central lesions such as degeneration of noradrenergic neurons in the locus coeruleus and hypofunction of striatal dopaminergic system may be the pathophysiological mechanism of PD sensory disturbances.^3^ Large amount of evidence has suggested that neuroinflammation and oxidative stress might play a vital role in the pathophysiological process of PD.^4^, ^5^ Specifically, triggering factors such as environmental exposure or age‐related conditions will lead to the activation of glia including microglia and astrocytes who serve as immune cells in central nervous system (CNS).^6^ Subsequently, inflammatory cytokines such as interleukin 1‐beta (IL‐1β), interleukin 6 (IL‐6), and tumor necrosis factor‐alpha (TNF‐α) will be secreted robustly as a “second hit” to cause more damage.^7^, ^8^, ^9^ Although extensive studies have focused on the central mechanisms of sensory disturbances and inflammatory processes in CNS, it is unclear whether the peripheral nervous system (PNS) is associated with these processes by far.

SCs are the most important glia in PNS, serving as supporting and protecting axons. Besides, they could also be activated into immune competent cells after peripheral nerve is injured and then produce inflammatory cytokines such as IL‐1β, IL‐6, and TNF‐α.^10^ In the last few decades, studies have reported structural and functional abnormalities in peripheral nerve from PD patients: loss of unmyelinated nerve fibers in sural nerve, increased thermal and tactile thresholds, reduced pain perception, and epidermal denervation.^11^, ^12^ Intriguingly, in our previous study, phosphorylated α‐synuclein (pSNCA) deposition, the classic pathological biomarker of PD, was found in Schwann cells (SCs) in sural nerve from PD patients using biopsy methods, demonstrating that SCs might be critically involved in peripheral nerve abnormalities in PD.^13^

Therefore, in this study, we hypothesized that Schwann cells activation and inflammatory cytokine release in peripheral sensory nerves of PD patients, which may lead to their sensory disturbances.

## 2 METHODS AND MATERIALS

### 2.1 Patients and controls

A total of 14 PD patients diagnosed according to the UK Parkinson's Disease Society Brain Bank criteria by two experienced neurologists were recruited at the Department of Neurology of the first affiliated hospital of Nanjing Medical University from November 2016 to December 2017.^14^ All enrollees should be idiopathic PD and voluntarily accept for sural nerve biopsy and sural nerve neurophysiological examination and there was no corresponding contraindication. Exclusive criteria included secondary Parkinsonism and atypical Parkinsonism consisting mainly of multiple system atrophy, corticobasal degeneration, progressive supranuclear palsy, and essential tremor. Patients without good response to levodopa treatment or have good response to levodopa but with significant cognitive dysfunction (mini‐mental state exam (MMSE) score < 24) were also excluded. Patients were not allowed to have a history of diabetes, alcoholism, chronic inflammatory demyelinating polyradiculoneuropathy, hereditary peripheral neuropathy, polyneuritis, HIV, syphilis, cancer, poliomyelitis, or abnormal laboratory tests that are predisposing causes for peripheral neuropathy (eg, fasting plasma glucose, glycosylated hemoglobin, fT3, fT4, TSH, serum vitamin B12 and folate, homocysteine, autoimmune antibodies). Motor symptoms were quantified using the Unified Parkinson's Disease Rating Scale (UPDRS) and the Hoehn and Yahr (H‐Y) staging. The mini‐mental state examination (MMSE) was applied to screen of global cognitive function.

We chose 6 age‐matched patients from the database of the Department of Pathology of the first affiliated hospital of Nanjing Medical University as controls who were suspicious of myopathy and accepted muscle and sural nerve biopsy, but were ultimately confirmed without neuropathy change. Also, these patients did not have a history of diabetes or other diseases that might damage the peripheral nerve, so these nerves were deemed as healthy controls. The demographic data for all participants were summarized in Table 1.

**Table 1:** Clinical characteristics of PD patients and controls

Case	Sex	Age	Disease duration (years)	UPDRS total score	Hoehn‐Yahr stage	LEDD (mg/d)	Sensory symptoms/More affected side/Symptom description
PD patient 1	M	73	2	25	2	600	−
PD patient 2	M	71	4	34	1	475	+/Right/Foot numbness
PD patient 3	M	59	3	60	1.5	400	+/Left/Calf soreness and pain
PD patient 4	M	72	1.5	41	1.5	300	−
PD patient 5	M	50	2	43	2.5	225	−
PD patient 6	M	65	1	41	1.5	500	−
PD patient 7	F	68	6	80	2.5	875	−
PD patient 8	F	62	3	42	2.5	450	−
PD patient 9	F	60	10	48	2.5	375	+/Right/Foot feeling of burning
PD patient 10	F	71	1.5	37	1	400	−
PD patient 11	F	60	3	49	2	600	+/Right/Foot numbness
PD patient 12	F	59	7	83	3	650	−
PD patient 13	F	67	4	80	2.5	800	−
PD patient 14	F	54	9	50	3	1000	+/Left/Calf soreness
Control 1	M	63	NS	NS	NS	NS	−
Control 2	M	59	NS	NS	NS	NS	−
Control 3	F	50	NS	NS	NS	NS	−
Control 4	F	65	NS	NS	NS	NS	−
Control 5	F	68	NS	NS	NS	NS	−
Control 6	F	71	NS	NS	NS	NS	−
PD patients (n = 14) Median (Range)	M = 6, F = 8	63.5 (50‐73)	3 (1‐10)	45.5 (25‐83)	2.25 (1‐3)	487.5 (225‐1000)	+ = 5, − = 9
Controls(n = 6)Median (Range)	M = 2, F = 4	64 (50‐71)	NS	NS	NS	NS	+ = 0, − = 6

Abbreviations: −, negative; +, positive; F, female; LEDD, levodopa equivalent daily dose; M, male; NS, no significance; PD, Parkinson's disease; UPDRS, Unified Parkinson's Disease Rating Scale.

This study was approved by the ethics committee of the First Affiliated Hospital of Nanjing Medical University, and all subjects have given their written informed consent before taking part in the study.

### 2.2 Electrophysiology

Nerve conduction velocity (NCV) and sensory nerve action potentials (SNAP) amplitudes of bilateral sural nerve from all subjects were completed in our laboratory by conventional surface electrodes using six channels electromyogram (Keypoint, Dantec Corporation). The history and physical examination of PD patients were performed by the formally trained neurologist prior to the electrophysiological examination (All subjects had no obvious abnormalities in muscle strength and muscle tone and pathological reflex was negative.). Results of NCV and SNAP amplitudes were classified as normal or abnormal based on our own laboratory collected age‐adjusted standardized database. In our study, when the age was ≥50 years, NCV reference range of sural nerve was >45.3 m/s, and SNAP amplitude reference range was >1.4 μV.

### 2.3 Sural nerve biopsy

Sural nerve biopsy was operated according to standardized procedures under local infiltration anesthesia. The more affected side of patients with sensory disturbances and the left side of patients without sensory disturbances were chosen. Fresh nerve specimens about 20‐30 mm in length and 2‐3 mm in diameter were dissected. 1‐mm pieces of nerve were cut and then fixed in glutaraldehyde for ultrathin sections. The remaining was fixed in 10% neutral formalin for 2 weeks, then dehydrated and embedded in paraffin. The tissue blocks were sectioned on a sliding microtome (Leica) at a thickness of 4 µm.

### 2.4 Immunohistochemistry and immunofluorescence

To analyze the expression of glial fibrillary acid protein (GFAP, a biomarker of Schwann cells) and cytokines, all tissue sections were dewaxed, rehydrated, and then incubated overnight at 4°C with one of the following primary antibodies: (a) a monoclonal mouse antibody against GFAP (GA5#3670, Cell Signaling Technology, 1:600) as a marker of the activation of Schwann cells; (b) a monoclonal mouse antibody against IL‐1β (sc‐52012, Santa Cruz Biotechnology, 1:50), a monoclonal mouse antibody against IL‐6 (sc‐130326, Santa Cruz Biotechnology, 1:300), and a monoclonal mouse antibody against TNF‐α (sc‐52746, Santa Cruz Biotechnology, 1:500). The next day, these sections were processed with corresponding secondary antibody and developed with diaminobenzene (DAKO; DAB). Sections were viewed with an LEICA DFC450 C microscope at magnification of ×10, ×20 and ×40. In each section, the immunostaining was scored semiquantitatively by two pathologists who were blinded to the patient information using a four‐point scale A (0 = absent, 1 = light yellow, 2 = light brown, 3 = dark brown) to determine the staining intensity and a five‐tier scale B (0 = none, 1 = 1%‐25%, 2 = 26%‐50%, 3 = 51%‐75%, 4 = 76%‐100%) as the positive range. The total score of each slice equals the product of scale A and B and take the average score of the two graders as the final result.^15^, ^16^

Double‐staining immunofluorescence was performed on four biopsies from PD patients to identify the cell types that were immonoreactive for cytokines. Recombinant rabbit monoclonal antibody to neurofilament (NF) heavy polypeptide (ab207176, Abcam, 1:1000) was used as the marker of axons, and recombinant rabbit monoclonal antibody to GFAP (ab33922, Abcam, 1:400) was used as the marker of Schwann cells. Sections were incubated in the combination of antibodies to NF with IL‐1β, IL‐6, and TNF‐α, and GFAP with IL‐1β, IL‐6, and TNF‐α, respectively, at 4°C overnight. Afterward, the sections were washed with PBS and then incubated with Alexa Fluor 488 (ab150077, Abcam, 1:400) and 594 (ab150108, Abcam, 1:1000) conjugated secondary antibodies for 2 hours at room temperature avoiding light. Slices were viewed under a fluorescent microscope (Zeiss) for colocalization images.

### 2.5 Electron microscopy

To characterize the morphological change of sural nerve, all specimens fixed in glutaraldehyde were incubated in 1% osmic acid for 2 hours in 4°C, then dehydrated in acetone and embedded in epon. For electron microscopy, specimens were cut into 70 nm thick sections and stained with uranyl acetate and lead citrate. Sections were observed under a FEI tecnai G2 SPIRIT microscope.

### 2.6 Statistical analysis

The SPSS 22.0 statistical package (IBM Corporation) was used to perform a normality test of the data. Quantitative data (such as the ages of PD patients and controls. The data obtained by Kolmogorov‐Smirnov test were normal: for PD patients, *P* > .20; for controls, *P* > .20) were compared using independent sample Student's *t* test; qualitative data (such as electrophysiological abnormality rate of PD patients with or without sensory disturbances) were compared using Fisher's exact test; since the data of four markers of immunohistochemistry were not normally distributed, we used Mann‐Whitney *U* test for comparison. A significance level of 5% was applied.

## 3 RESULTS

### 3.1 The demographic and clinical characteristics

The clinical data were summarized in Table 1. In total, 14 idiopathic PD patients and 6 controls were included in this study. There was no significant difference in the age of PD patients compared with controls (Mean ± SD: PD patients 63.6 ± 7.1, controls 62.7 ± 7.4, independent sample *t* test *P* = .784). PD‐related sensory symptoms were reported in 5 out of 14 patients as shown in Table 1.

### 3.2 Electrophysiology

Electrophysiological results were shown in Table 2. Seven of 14 PD patients had abnormalities in unilateral or bilateral sural nerve electrophysiological examination results, but 0/6 controls had and the difference was statistically significant (*P* = .044). Four of 5 PD patients with sensory disturbances and 3/9 PD patients without sensory disturbances had abnormalities in unilateral or bilateral sural nerve electrophysiological examination results. There was no significant difference in the positive rate of abnormal sural nerve electrophysiological results between PD patients with and without sensory disturbances (*P* = .266). Therefore, the subjective sensory disturbances of the lower limbs of PD patients were not completely consistent with the sural nerve electrophysiological results.

**Table 2:** Sural nerve electrophysiological examination results of PD patients and controls

Case	Sensory disturbance	Sex	Age	Left side SNAP amplitude (μV)	Right side SNAP amplitude (μV)	Left side NCV (m/s)	Right side NCV (m/s)
PD patient 1	+	F	54	0	2.3	0	53.8
PD patient 2	+	M	59	0	0	0	0
PD patient 3	+	F	60	12	9.1	59.5	55.8
PD patient 4	+	F	60	0	0	0	0
PD patient 5	+	M	71	0	0	0	0
PD patient 6	−	F	67	6.8	5	48.8	44
PD patient 7	−	F	71	0	0	0	0
PD patient 8	−	M	65	6.8	7.2	46.3	51.9
PD patient 9	−	M	72	5.3	6.3	59.5	63.5
PD patient 10	−	M	73	8.6	11.7	54.3	51.5
PD patient 11	−	F	62	0	0	0	0
PD patient 12	−	F	68	12.7	5.6	69.4	63.8
PD patient 13	−	F	59	5.6	6.3	76.9	65.3
PD patient 14	−	M	50	6	6.2	59.5	63.3
Control 1	−	M	63	5.1	7	82.5	69.9
Control 2	−	M	59	8.9	17.6	56	46
Control 3	−	F	50	15.4	11.9	65.5	72.1
Control 4	−	F	65	6.4	4.5	63.7	58
Control 5	−	F	68	5.5	9.6	52.6	50.9
Control 6	−	F	71	22.8	7.1	62	63.7

Abbreviations: −, negative; +, positive; F, female; M, male; PD, Parkinson's disease; SNAP, sensory nerve action potential; NCV, nerve conduction velocity.

### 3.3 Immunohistochemistry and immunofluorescence

**Figure 1:**
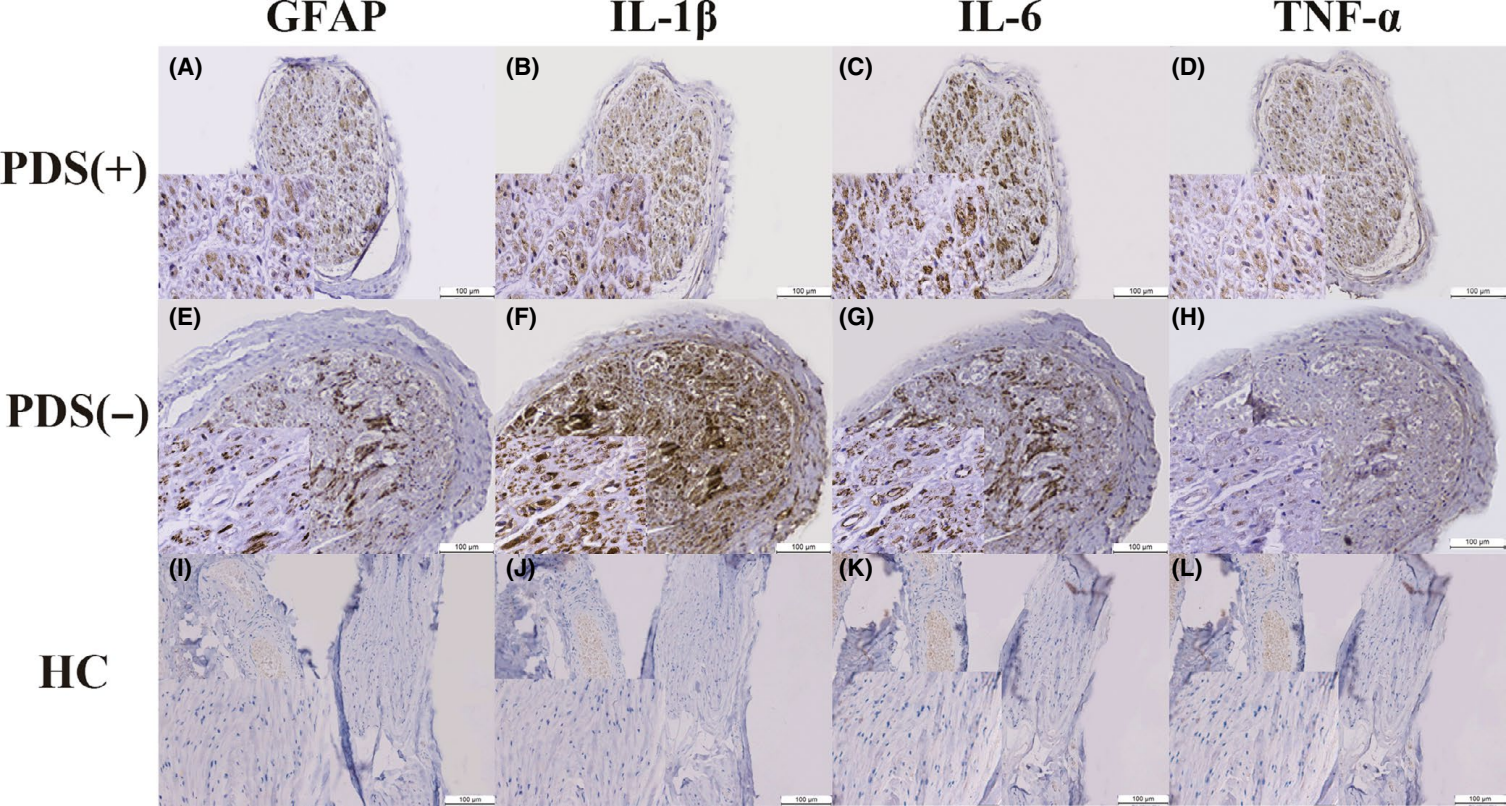
GFAP, IL‐1β, IL‐6, and TNF‐α in serial sections of sural nerve. The brown inclusions inside the nerve bundles were positive immunoreaction to GFAP (A, E, I), IL‐1β (B, F, J), IL‐6(C, G, K), and TNF‐α (D, H, L) in a 60‐y‐old female PD patient with sensory disturbances (A‐D) (case PD patient 11 in Table 1), a 71‐y‐old female PD patient without sensory disturbances (E‐H) (case PD patient 10 in Table 1), and a 63‐y‐old male control without neuropathy (I‐L) (case Control 1 in Table 1). Original magnification was 100×. Insets were all set at magnification (400×). *Abbreviations*: GFAP, glial fibrillary acid protein; HC, healthy controls; IL‐1β, interleukin‐1‐beta; IL‐6, interleukin‐6; PD, Parkinson's disease; PDS (−), PD patient without sensory disturbances; PDS (+), PD patient with sensory disturbances; TNF‐α, tumor necrosis factor‐alpha

Sections were viewed by two pathologists who were blind to the information. With a quick view, controls had low reactivity with GFAP and three inflammatory factors IL‐1β, IL‐6, and TNF‐α. In contrast, 14 PD samples showed increased expression of GFAP and inflammatory cytokines (Figure 1).

**Figure 2:**
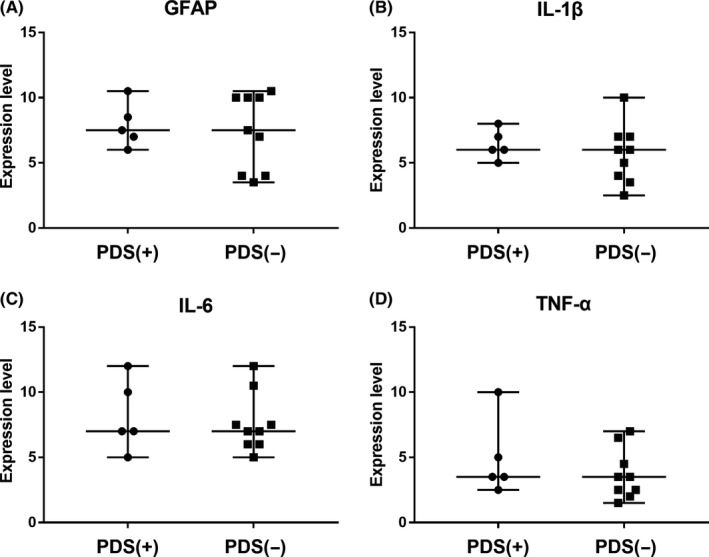
Expression levels of GFAP, IL‐1β, IL‐6, and TNF‐α in sural nerve biopsies from PD patients with sensory disturbances (round) and without disturbances (square). There was no difference in the levels of GFAP, IL‐1β, IL‐6, and TNF‐α between PD patients with and without sensory disturbances (*P* = .797, .438, .898, and .438, respectively). Horizontal bar represented median and range. Data were analyzed using Mann‐Whitney *U* test. *Abbreviations*: GFAP, glial fibrillary acid protein; IL‐1β, interleukin‐1‐beta; IL‐6, interleukin‐6; PDS (−), PD patient without sensory disturbances; PDS (+), PD patient with sensory disturbances; TNF‐α, tumor necrosis factor‐alpha

To test if the expression of cytokines were associated with sensory disturbances, we set aside controls and analyzed the results of PD patients alone. We divided PD patients into two groups according to whether they had sensory symptoms or not and compared the expression of both GFAP and cytokines. We found no difference in the expression levels of GFAP and the three inflammatory factors IL‐1β, IL‐6, and TNF‐α between patients with and without sensory symptoms. (*P* = .797, .438, .898, and .438, respectively) (Figure 2).

**Figure 3:**
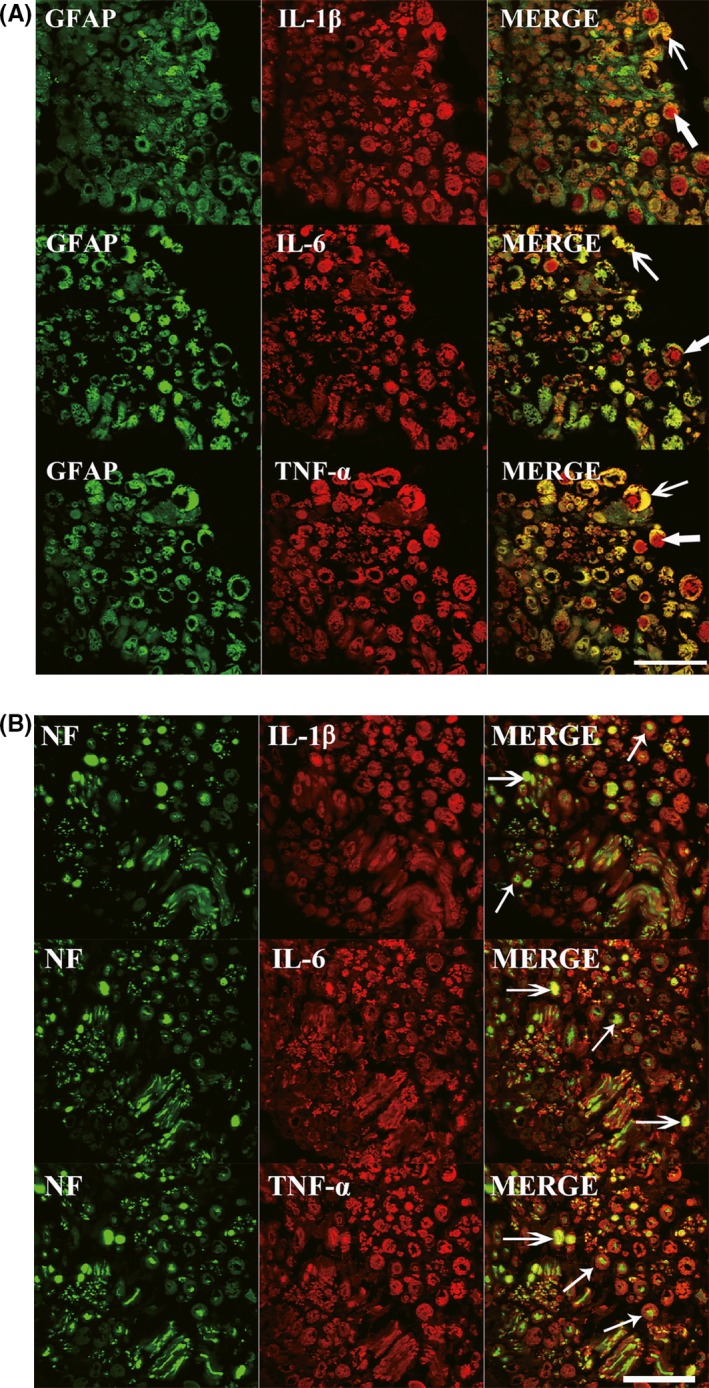
Double‐staining immunofluorescence for cytokines with GFAP and NF in sural nerve from a PD patient (case PD patient 10 in Table 1). A, Immunostaining for GFAP with TNF‐α, IL‐1β, and IL‐6. Partial cytokines was colocalized with Schwann cells as indicated by *thin arrows* in A, and the rest cytokines were surrounded by the colocalization as indicated by *thick arrows*. B, Immunostaining for NF with TNF‐α, IL‐1β, and IL‐6. *Thick arrows* indicate cytokines colocalized with axons, and *thin arrows* indicate cytokines surround axons. Bar 50 μm. *Abbreviations*: GFAP, glial fibrillary acid protein; IL‐1β, interleukin‐1‐beta; IL‐6, interleukin‐6; NF, neurofilament; PD, Parkinson's disease; TNF‐α, tumor necrosis factor‐alpha

The results of double‐staining immunofluorescence showed that cytokines were remarkably colocalized with GFAP and slightly with NF (Figure 3), indicating that both Schwann cells and axons were immunoreactive for the three cytokines and Schwann cells are more reactive.

### 3.4 Electron microscopy

**Figure 4:**
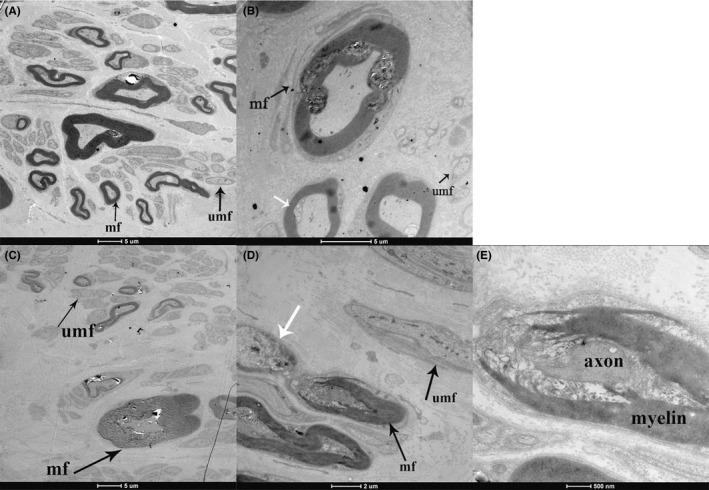
Electron photomicrographs of sural nerve fibers from a control (Figure 4A), a PD patient with sensory disturbances (Figure 4B) and a PD patient without sensory disturbances (Figure 4C‐E). A, Normal sural nerve from a control (case Control 2 in Table 1); (B) sural nerve from a PD patient with sensory disturbances (case PD patient 3 in Table 1). C‐E, sural nerve from a PD patient without sensory disturbances with different magnifications (case PD patient 9 in Table 1). A, In normal sural nerve, myelinated and unmyelinated fibers are distributed evenly with uniform size. B and C, The abnormality in PD is fewer myelinated and unmyelinated fibers than control. B and D, The foam‐like axon indicated by the white arrow may represent axonal degeneration. B‐E, The myelin is swollen and partly degrades into debris, and the axon‐Schwann cell contact is therefore damaged. “mf” as myelinated fibers and “umf” as unmyelinated fibers

The electron photomicrographs clearly showed both myelinated and unmyelinated fibers of sural nerve from PD patients and controls. In addition, we observed the debris and foam‐like body inside the axons in the sural nerve of PD patients with or without sensory disturbances, demonstrating that the axons were degenerating (white arrow in Figure 4B and D); the swollen myelin and fragmentized subunit of Schwann cells indicated demyelination of sural nerve of PD (Figure 4B‐D).

## 4 DISCUSSION

Most of the existing studies center around the central mechanism of PD sensory disturbances and the inflammatory process of CNS. Our study focused on peripheral nerve inflammation and sought to find peripheral mechanisms of sensory disturbances. Sural nerve is composed of the axons of primary sensory neurons conducting feelings mainly from the lower limbs and Schwann cells sheathing around axons. In this study, analyzing sural nerve from patients with PD and controls, we obtained the following findings. First, the nerve conduction velocity (NCV) slowed down and the sensory nerve action potential (SNAP) amplitude decreased in PD patients compared to healthy controls. This indicated that conduction function of sural nerve was impaired and the peripheral sensory nerve injury in PD patients was definitely present. However, there was no significant difference in electrophysiological findings between PD patients with or without sensory impairment. The possible reasons for this result are as follows: (a) The sample size is small; (b) it is failed to show the abnormal nerve conduction above the sural nerve; (c) the changes in fine fibers could not be detected by electrophysiological testing. So the subjective sensory abnormalities of the lower limbs of PD patients were not completely consistent with the electrophysiological results of sural nerve. Secondly, more importantly, the expression of GFAP and inflammatory cytokines IL‐1β, IL‐6, and TNF‐α were increased both in PD patients with and without sensory impairment. And obtained by semiquantitative immunohistochemistry, there was no significant difference in the expression of the four indicators between the two groups. The expression of control samples was very low. This indicated that Schwann cells activation and inflammatory response of peripheral sensory nerves in PD patients existed exactly, but there was no necessary connection with sensory disturbances. This result may be due to the following: the sample size is small; sensory disturbances may come from central lesions, sensory disturbances are subjective experiences and susceptible to psychological factors, or semiquantitative analysis itself has errors, etc In addition, the results of double immunofluorescence identified that both the Schwann cells and axons were immunoreactive for the three cytokines. Ultrastructure changes of peripheral nerve from PD patients including degenerating axons and demyelination were observed in electron micrographs. These were present and did not differ in PD patients with and without sensory impairment. This indicated there was a certain correlation between neuroinflammation and axonal degeneration and demyelinating lesions in PD patients, but there was no significant correlation between neuroinflammation and subjective sensory disturbances.

Schwann cells are considered the peripheral counterpart of glia in CNS, functioning as supporting and protecting axons, and contributing to the regeneration of injured neurons and axons via the axon‐Schwann cell contact.^17^, ^18^, ^19^ Besides, Schwann cells could be activated and turned into immune competent cells when an insult occurs, and the expression of GFAP is correlated with the severity of inflammation.^10^ In our study, Schwann cells were activated accompanied with elevated inflammatory cytokines, and the results of double immunofluorescence showed that cytokines were detected not only in Schwann cells but also in axons. Moreover, ultrastructural detection revealed axonal degeneration of the nerve and axonal‐Schwann cell contact dysfunction. It could be inferred that inflammatory reaction may contribute to both axonal degeneration and axon‐Schwann cell contact malfunctions, suggesting neuroinflammation may be involved in the pathogenesis of peripheral neuropathy in PD.

Studies have found many PD patients have sensory disturbances such as pain and lower limb numbness.^1^, ^2^ More importantly, these symptoms could not be remitted by levodopa, suggesting a different underlying pathophysiological process from that in substantia nigra.^20^ Peripheral inflammation was found to be involved in many symptoms in PD. For example, similar results with ours were observed in the gut of PD patients where elevated GFAP and cytokines including IL‐1β, IL‐6, and TNF‐α were detected, which implied that enteric inflammation might play a role in the intestinal tract change in PD patients.^21^ Therefore, it was reasonable to speculate that inflammation present in the peripheral somatosensory nerve may cause these sensory disturbances. However, there was no difference in the expression of GFAP and inflammatory cytokines between PD patients with and without sensory disturbances. Therefore, we speculated peripheral inflammation was not necessarily related to sensory symptoms. Because Sumikura et al found pSNCA in the dorsal spinal horn and the dorsal root ganglia (DRG) in PD patients which may also lead to paresthesia.^22^

There is no controversy that inflammation exists in PD and our study just extends this knowledge to primary somatic sensory afferent nerve. There are some limitations in our study. Firstly, the sample size is relatively small which weakens the power of the statistic. Secondly, this is a cross‐sectional study in which we are not able to analyze the correlation between the cytokines and the disease progression of PD patients. Thirdly, we failed to quantify the number of sural nerve fibers and evaluating the number of Schwann cells and axons in all samples could bring more convincing results to our study. And we did not explore in depth the possible difference between SCs with myelinated nerve fibers and SCs with unmyelinated nerve fibers. We hope further work could overcome these limitations and dig more information from peripheral nerve in PD.

## 5 CONCLUSION

In conclusion, our study results suggest that peripheral sensory nerve injury exists in PD patients, accompanied by Schwann cell activation and inflammation, thus demonstrate peripheral nerve inflammation participates in the pathophysiological process of PD and this may be related to sensory disturbances of PD patients but not necessarily related.

## CONFLICT OF INTEREST

None.
